# Investigating the Origins of Biochemical Dysregulation in Trisomy 21 Pregnancy

**DOI:** 10.1210/clinem/dgaf339

**Published:** 2025-06-09

**Authors:** Angelika Buczyńska, Iwona Sidorkiewicz, Adam Jacek Krętowski, Monika Zbucka-Krętowska

**Affiliations:** Clinical Research Centre, Medical University of Białystok, Białystok 15–276, Poland; Clinical Research Support Centre, Medical University of Białystok, Białystok 15–276, Poland; Clinical Research Centre, Medical University of Białystok, Białystok 15–276, Poland; Department of Endocrinology, Diabetology and Internal Medicine, Medical University of Białystok, Białystok 15–276, Poland; Department of Gynecological Endocrinology and Adolescent Gynecology, Medical University of Białystok, Białystok 15–276, Poland

**Keywords:** trisomy 21, oxidative stress, antioxidant capacity, leptin, adiponectin, sirtuin 1

## Abstract

**Background and Aims:**

Genetic profiling of trisomy 21 (T21) shows disruptions in energy homeostasis and oxidative stress pathways. This study aimed to evaluate oxidative and metabolic dysregulation in T21 pregnancies and identify their genetic or systemic origins.

**Methods:**

After karyotype analysis, 20 women with T21 and 20 with euploid fetuses were enrolled. Inclusion criteria were referral for prenatal testing; exclusion criteria encompassed maternal chronic or acute diseases. Amniotic fluid and plasma samples were collected and total oxidative capacity (TOC), total antioxidant capacity (TAC), superoxide dismutase (SOD), nuclear factor-κB (NFκB), forkhead box O (FOXO), sirtuin 1 (SIRT1), 8-hydroxy-2′-deoxyguanosine (8-OHdG), leptin, and adiponectin levels were measured. The amniotic fluid/plasma (AF/P) ratio was calculated to assess marker origin.

**Results:**

In T21 pregnancies, maternal plasma showed decreased levels of TAC, SOD, FOXO, and leptin (*P* < .05). In amniotic fluid, levels of FOXO and leptin were also reduced, while SOD, TAC, TOC, adiponectin, and 8-OHdG were elevated (*P* < .05). The AF/P ratio was increased for SOD (*P* = .027), TAC (*P* < .001), TOC (*P* < .0001), and adiponectin (*P* = .002), suggesting a fetal origin, while decreased SIRT1 levels (*P* = .036) indicate impaired fetal oxidative regulation. A plasma biomarker panel comprising SOD, TAC, and leptin, assessed via regression modeling, demonstrated the highest clinical utility in distinguishing T21 pregnancies from euploid pregnancies (area under the receiver operating characteristic curve = .92, *P* < .001).

**Conclusion:**

The AF/P ratio supports a fetal origin for SOD, TAC, TOC, and adiponectin, while lower SIRT1 implies disrupted fetal oxidative regulation.

Trisomy 21 (T21), which appears in approximately 1 in 700 live births worldwide, is an autosomal aneuploidy that manifests as the Down syndrome (DS) phenotype (1). This chromosomal aberration results from the presence of an extra copy of chromosome 21, arising due to insufficient chromosome segregation during gametogenesis, as observed in approximately 95% of affected individuals (2). A meta-analysis of gene expression patterns in DS patients reveals significant alterations in mitochondrial and bioenergetic pathways, as well as disruptions in lipid metabolism and oxidative stress responses, indicating a state of redox imbalance (3-6). Oxidative stress during pregnancy may exacerbate developmental anomalies by inducing DNA damage, cellular apoptosis, and compromised placental function, thereby perturbing crucial cellular processes essential for normal fetal growth, organogenesis, and maturation (7, 8). Given the complex nature of T21 pathogenesis, it is essential to evaluate molecules related to specific T21 gene patterns that may influence fetal development within both maternal and fetal compartments to understand the origin and interdependencies between the mother and fetus (3, 9-17). Oxidative imbalance could be expressed through total oxidant capacity (TOC), with 8-hydroxy-2′-deoxyguanosine (8-OHdG) commonly used to indicate DNA damage and decreased total antioxidant capacity (TAC) (7, 18-21). Superoxide dismutase (SOD) plays a crucial role in maintaining redox balance by scavenging superoxide radicals, while nuclear factor-κB (NFκB) and forkhead box O (FOXO) are key regulators of oxidative stress responses, highlighting the importance of these pathways in managing oxidative imbalance (22-25). Additionally, sirtuin 1 (SIRT1) ensures genome stability and oxidative homeostasis, and leptin and adiponectin further contribute by activating antioxidant pathways to reduce oxidative stress and regulate energy metabolism (26-31). These specific markers were selected for analysis due to their well-established roles in oxidative stress, DNA damage repair, and metabolic regulation, all of which are likely to be disrupted in the pathogenesis of T21 and may contribute to its underlying biochemical dysregulation. Additionally, increased oxidative stress elevates the risk of preterm birth, underscoring the imperative for novel management strategies to support fetal development. This is particularly crucial for pregnant women carrying fetuses with T21, as increased oxidative stress has been demonstrated by their early outcomes and specific DS gene patterns (9, 16, 17).

The objective of this study is to initially evaluate key biochemical markers associated with oxidative stress and metabolic dysregulation in both maternal and fetal compartments in pregnancies affected by T21. Subsequently, the study aims to determine the origin of these markers—whether they are directly related to the gene expression changes caused by the presence of an additional chromosome 21 or are secondary to systemic physiological responses during T21 pregnancies. By elucidating the relationship between these biochemical markers and the gene expression profiles specific to T21 pathogenesis, this study aims to establish refined screening panels and contribute to the development of personalized clinical management strategies for T21 pregnancies.

## Material and Methods

### Participant Recruitment

This prospective case-control study involved women undergoing routine amniocentesis between the 15th and 18th weeks of gestation at the Department of Reproduction and Gynecological Endocrinology, Medical University of Bialystok, Poland, from 2017 to 2020. From an initial cohort of 100 pregnant women screened, 40 were enrolled in the study based on defined eligibility criteria, including the absence of chronic conditions (such as hypertension, diabetes, or autoimmune disorders) and acute illnesses (such as active infections or recent surgical interventions). Indications for amniocentesis included an elevated risk of chromosomal abnormalities in noninvasive prenatal screening and maternal age over 35 years. Exclusion criteria encompassed the presence of chronic or acute illnesses such as gestational or pre-existing diabetes mellitus, chronic hypertension, preeclampsia, autoimmune diseases (eg, systemic lupus erythematosus, Hashimoto's thyroiditis), thyroid dysfunction, renal impairment, cardiovascular disease, active infections, and recent surgical interventions. Additional exclusion criteria included current use of hormonal or anti-inflammatory medications, high-risk pregnancy status, and a history of preterm delivery. All participants received comprehensive information about the study and the potential risks associated with amniocentesis. Amniotic fluid samples with potential blood contamination were excluded from the study. Power analysis was employed to determine the appropriate sample size for detecting significant differences in the studied parameters between groups. With a 5% margin of error and 95% confidence level, the recommended sample size of study group for our preliminary research was 16 (32). Following karyotype analysis, 20 women carrying fetuses with T21 and 20 women with euploid fetuses were selected for the study. The study group exhibited no significant differences in age, pregnancy progression, or body mass index. Fasting blood (5.5 mL) and amniotic fluid samples were collected from all participants on the day of amniocentesis. Biological material underwent centrifugation for plasma separation and was subsequently stored at −80 °C.

### Ethical Considerations

This study was conducted in accordance with the Declaration of Helsinki and was approved by the Ethics Committee of the Medical University of Bialystok, Poland (APK/002/351/2020). Informed consent was obtained from all subjects involved in the study.

### Laboratory Analyses

The TOS status was assessed using photometric immunodiagnostic assay [PerOx (TOS/TOC) Kit, KC5100, 64625 Bensheim, Germany] and the TAC status was assessed by photometric assay [ImAnOx (TAS/TAC) Kit, KC5200, 64625 Bensheim, Germany]. NFκB, FOXO, SIRT1, and 8-OhDG concentrations were determined using an ELISA (Enzyme-linked Immunosorbent Assay Kit; Cloud-Clone Corp., Wuhan, China; Cloud-Clone Corp Cat# SEB824Hu, RRID:AB_3696578; Cloud-Clone Corp Cat# SEA764Hu, RRID:AB_3696580; Cloud-Clone Corp Cat# SEE912Hu, RRID:AB_3696581; Cloud-Clone Corp Cat# CEA660Ge, RRID:AB_3696583, respectively) according to the manufacturer's instructions. SOD activity was measured using colorimetric activity method (KO28-H1, Ann Arbor, MI, USA). The concentration of the metabolic-related markers, such as leptin (BioVendor Cat# RD191001100, RRID:AB_3696585, Inc., Modrice, Czech Republic) and adiponectin (Millipore Cat# EZHADP-61 K, RRID:AB_2801457, Millipore Corporation, Billerica, MA USA) were also measured using ELISA methods. Samples and controls (plasma and amniotic fluid) were analyzed together using a blind analysis approach. The amniotic fluid/plasma ratio (AF/P) was calculated to assess the potential fetal or maternal origin of marker alterations.

### Statistical Analysis

Statistical analyses were carried out with GraphPad Prism v. 9.0 (GraphPad Software, Inc., San Diego, CA, USA). The normality of data distribution was assessed using the Shapiro–Wilk test, indicating nonnormal distribution. Consequently, group comparisons were conducted using the nonparametric Mann–Whitney test, with *P* < .05 considered significant. To account for the risk of type I error due to multiple comparisons, the Bonferroni correction was applied where appropriate. Correlation analyses between the concentrations of all studied parameters in plasma and amniotic fluid samples were performed using the Spearman test for multiple comparisons. The receiver operating characteristic (ROC) curves were generated with simultaneous sensitivity and specificity calculations, and area under the ROC curve (AUC) greater than 0.50 was considered significant (when *P* < .05). Furthermore, to determine whether the oxidative stress markers are derived from T21 itself or are a systemic phenomenon resulting from peripheral processes, logistic regression analysis was conducted. During the feature selection phase, we identified plasma biomarkers with the highest predictive power, selecting those with the lowest corrected Akaike information criterion and minimal multicollinearity, as assessed by variance inflation factor and R2 with other variables evaluations. Moreover, the panel was checked by the Hosmer-Lemeshow test.

### Validation Design

The study assessed the origin of biochemical markers by determining whether they are directly associated with gene expression changes due to the presence of an additional chromosome 21 or are a result of systemic physiological responses during T21 pregnancies. This was accomplished using logistic regression analysis alongside an exploration of genetic databases. The genetic pattern related to T21 was determined using the Genevestigator program, which analyzes genetic data from single-cell RNA sequencing. This study utilized available databases. Appropriate cell-specific features collected from amniotic fluid samples from Genevestigator databases were selected, focusing on data collected from fetuses with T21. Genevestigator was used to analyze gene expression data, specifically targeting genes responsible for all cellular responses to oxidative stress in T21 fetuses. The results of the analyses were compared with existing literature to confirm their significance and consistency with previously described studies.

## Results

### Plasma Measurement

**Figure 1.**
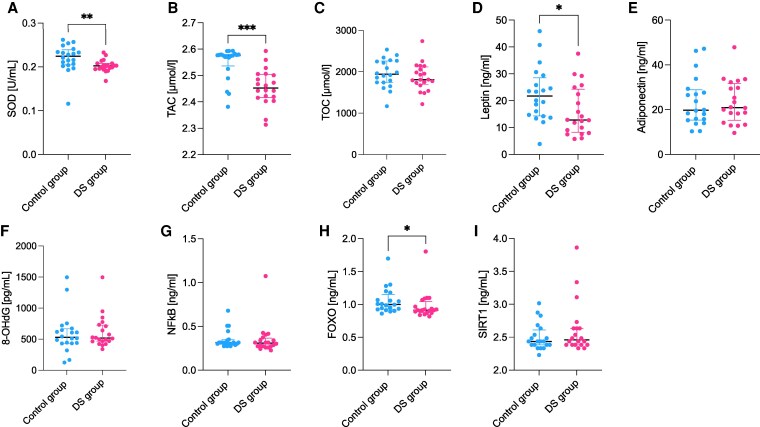
The plasma oxidative stress and metabolic markers profiling where results are presented in median and interquartile range (* *P* ≤ .05, ** *P* ≤ .01, *** *P* ≤ .001), with *P* < .05 considered significant. (A) SOD; (B) TAC; (C) TOC; (D) leptin; (E) adiponectin; (F) 8-OHdG; (G) NFκB; (H) FOXO; (I) SIRT1.

Abbreviations: 8-OHdG, 8-hydroxy-2′-deoxyguanosine; AF, amniotic fluid; FOXO, forkhead box protein O; NFκB, nuclear factor kappa-light-chain-enhancer of activated B cells; *P, P*-value; SIRT1, sirtuin 1; SOD, superoxide dismutase; TAC, total antioxidant capacity; TOC, total oxidative capacity.

During the analysis of oxidative stress-related biomarkers in maternal plasma, the activity of SOD and TAC levels were observed to be decreased in the T21 group compared to the control group (*P* = .003, *P* < .001, respectively). Additionally, FOXO levels were decreased in the T21 group (*P* = .034). Furthermore, the plasma leptin concentrations were also found to be lower in the T21 group when compared to the controls (*P* = .029) (Fig. 1).

### Assessments in Amniotic Fluid Samples

**Figure 2.**
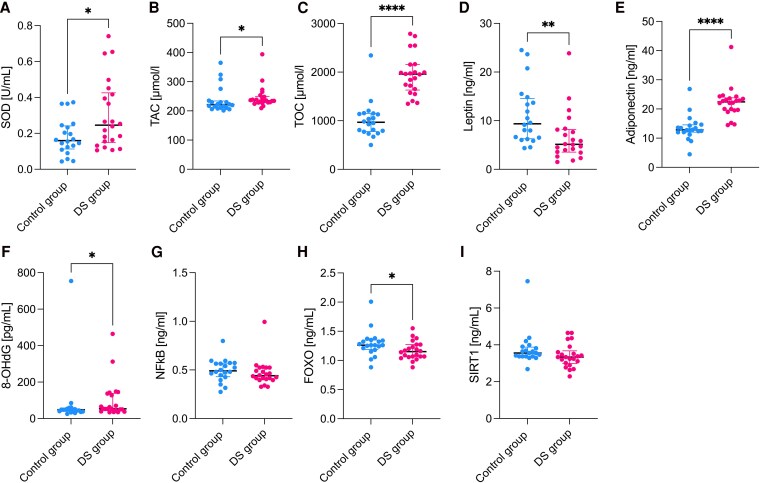
The AF oxidative stress and metabolic markers profiling where results are presented in median and interquartile range (* *P* ≤ .05, ** *P* ≤ .01, **** *P* ≤ .0001), with *P* < .05 considered significant. (A) SOD; (B) TAC; (C) TOC; (D) leptin; (E) adiponectin; (F) 8-OHdG; (G ) NFκB; (H) FOXO; (I) SIRT1.

Abbreviations: 8-OHdG, 8-hydroxy-2′-deoxyguanosine; AF, amniotic fluid; FOXO, forkhead box protein O; NFκB, nuclear factor kappa-light-chain-enhancer of activated B cells; *P, P*-value; SIRT1, sirtuin 1; SOD, superoxide dismutase; TAC, total antioxidant capacity; TOC, total oxidative capacity.

The parameters in the amniotic fluid were evaluated to assess the processes occurring in the fetal compartment. The analysis of oxidative stress-related markers in amniotic fluid showed that the activity of SOD, levels of TAC and TOC, and 8-OhDG concentration were increased in the T21 group compared to the control group (*P* = .033, *P* = .020, *P* < .001, *P* = .038, respectively). Additionally, FOXO levels were lower in the T21 group compared to controls (*P* = .044). Furthermore, the amniotic fluid's concentrations of leptin were also lower in the T21 group compared to the controls (*P* = .004). Interestingly, the adiponectin concentrations were increased among T21 group comparing to controls (*P* < .001) (Fig. 2).

### Correlation

**Figure 3.**
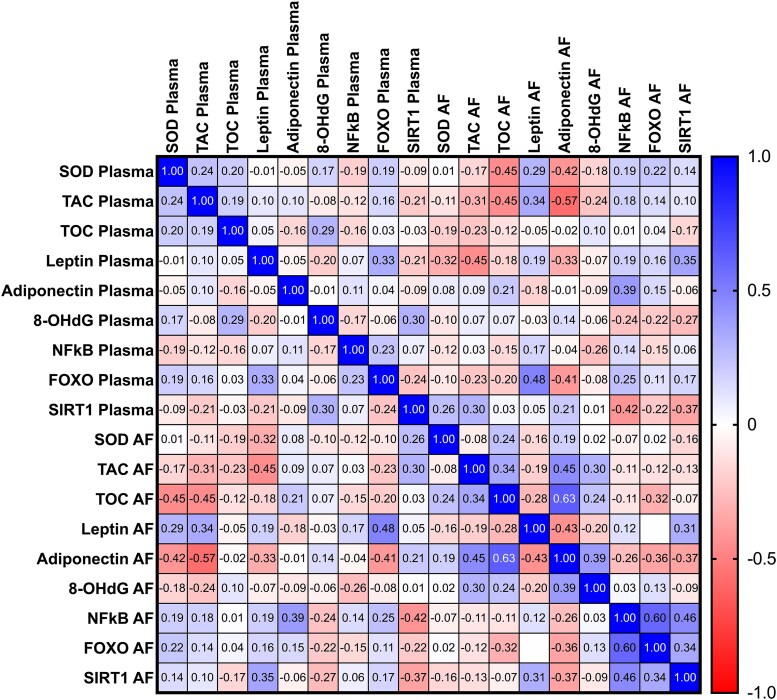
The correlation matrix.

Abbreviations: 8-OHdG, 8-hydroxy-2′-deoxyguanosine; AF, amniotic fluid; FOXO, forkhead box protein O; NFκB, nuclear factor kappa-light-chain-enhancer of activated B cells; SIRT1, sirtuin 1; SOD, superoxide dismutase; TAC, total antioxidant capacity; TOC, total oxidative capacity.

Moreover, Spearman correlation was evaluated to assess the relationships between the studied parameters (Fig. 3) within the whole group. During the analysis of SIRT1 plasma concentration, negative correlations were observed between plasma NFκB and SIRT1 levels (r = −0.42, *P* < .05). Additionally, a negative correlation was found between plasma SOD activity and amniotic fluid adiponectin levels (r = −0.42, *P* < .01) and between plasma TAS/TAC and amniotic fluid adiponectin levels (r = −0.57, *P* < .001). Furthermore, positive correlations were identified between plasma TAS/TAC and amniotic fluid leptin (r = 0.34, *P* < .05), as well as between plasma leptin and plasma FOXO (r = 0.32, *P* < .05) and amniotic fluid SIRT1 (r = 0.35, *P* < .05) concentrations. Moreover, a negative correlation was observed between plasma leptin and SOD activity (r = −0.33, *P* < .05), TAS/TAC (r = −0.45, *P* < .01), and adiponectin (r = −0.33; *P* < .05) levels measured in amniotic fluid. Conversely, a positive correlation was demonstrated between plasma FOXO and plasma and amniotic fluid leptin levels (r = 0.33, r = 0.48; all *P* < .05, respectively). The negative correlation between plasma FOXO and amniotic fluid adiponectin levels (r = −0.4, *P* < .05) was also observed (Fig. 3).

### Comparison of the Amniotic Fluid-to-plasma Ratio

**Figure 4.**
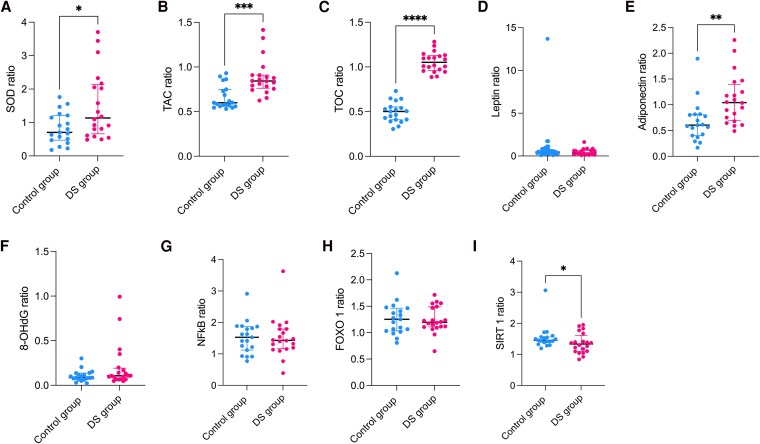
The AF/plasma ratio evaluation between studied groups where results are presented in median and interquartile range (* *P* ≤ .05, ** *P* ≤ .01, **** *P* ≤ .0001), with *P* < .05 considered significant. **(**A) SOD; (B) TAC; (C) TOC; (D) leptin; (E) adiponectin; (F) 8-OHdG; (G) NFκB; (H) FOXO; (I) SIRT1.

Abbreviations: 8-OHdG, 8-hydroxy-2′-deoxyguanosine; AF, amniotic fluid; FOXO, forkhead box protein O; NFκB, nuclear factor kappa-light-chain-enhancer of activated B cells; *P, P*-value; SIRT1, sirtuin 1; SOD, superoxide dismutase; TAC, total antioxidant capacity; TOC, total oxidative capacity.

To assess intergroup differences and investigate whether the observed metabolic disturbances originate from maternal or fetal origin or constitute a physiological response, we calculated the AF/P ratio. This was determined by dividing the concentrations measured in amniotic fluid by those obtained in maternal plasma. This analytical approach facilitates the evaluation of potential alterations in biosynthesis and transport mechanisms across the maternal-fetal interface. The obtained results demonstrated that the AF/P ratio was significantly elevated in the study group compared to the control group for SOD (*P* = .027), TAC (*P* < .001), TOC (*P* < .0001), and adiponectin (*P* = .002), whereas a significant decrease was observed for SIRT1 (*P* = .036) (Fig. 4).

### Clinical Utility Based on ROC Evaluation

#### Plasma

**Figure 5.**
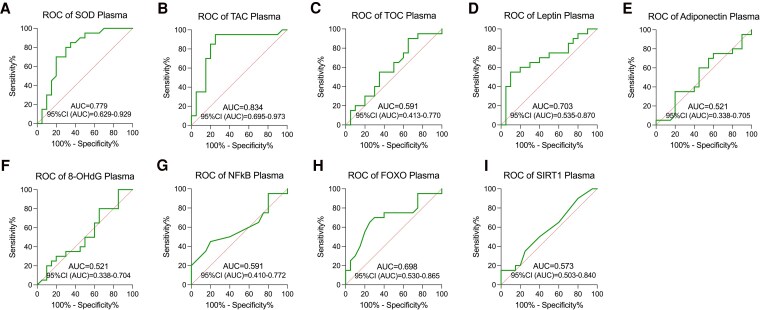
The ROC curves of plasma protein, with *P* < .05 considered significant. (A) SOD; (B) TAC; (C) TOC; (D) leptin; (E) adiponectin; (F) 8-OHdG; (G) NFκB; (H) FOXO; (I) SIRT1.

Abbreviations: 8-OHdG, 8-hydroxy-2′-deoxyguanosine; AUC, area under the ROC curve; FOXO, forkhead box protein O; NFκB, nuclear factor kappa-light-chain-enhancer of activated B cells; *P, P*-value; ROC, receiver operating characteristic; SIRT1, sirtuin 1; SOD, superoxide dismutase; TAC, total antioxidant capacity; TOC, total oxidative capacity.

To assess the significance of oxidative stress and metabolic markers in T21 screening, we conducted ROC curve analysis. However, the screening effectiveness of TOS/TOC, adiponectin, 8-OHdG, NFκB, and SIRT1 was not evaluated as they did not differ between T21 and control groups (*P* > .05). Among the differentially expressed markers, TAS/TAC demonstrated the highest screening utility (AUC = 0.83), followed by SOD (AUC = 0.78), FOXO (AUC = 0.70), and leptin (AUC = 0.70) (all parameters, *P* < .05) (Fig. 5).

#### Amniotic fluid

**Figure 6.**
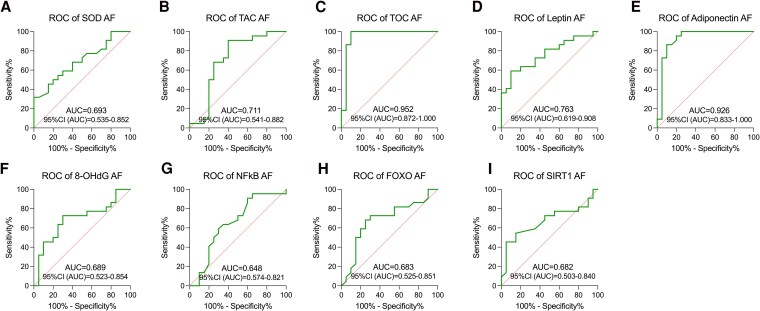
The ROC curves of amniotic fluid protein, with *P* < .05 considered significant. (A) SOD; (B) TAC; (C) TOC; (D) leptin; (E) adiponectin; (F) 8-OHdG; (G) NFκB; (H) FOXO; (I) SIRT1.

Abbreviations: 8-OHdG, 8-hydroxy-2′-deoxyguanosine; AUC, area under the ROC curve; FOXO, forkhead box protein O; NFκB, nuclear factor kappa-light-chain-enhancer of activated B cells; *P, P*-value; ROC, receiver operating characteristic; SIRT1, sirtuin 1; SOD, superoxide dismutase; TAC, total antioxidant capacity; TOC, total oxidative capacity.

We also conducted an analysis of the screening utility of oxidative stress markers and metabolic markers in amniotic fluid. The screening effectiveness of NFκB and SIRT1 was not assessed following no differences observed between groups (*P* > .05). Analyzing the AUC, the highest screening value was determined for TOS/TOC (AUC = 0.95) and adiponectin (AUC = 0.93), followed by leptin (AUC = 0.76), TAS/TAC (AUC = 0.71), SOD, and 8-OHdG (both AUC = 0.69). The screening utility for amniotic fluid FOXO (AUC =0.68) was the lowest among all studied biomarkers (*P* < .05) (Fig. 6).

#### Odds ratio evaluation

Furthermore, to determine the origin of oxidative stress and metabolic markers, precisely whether they arise directly from the T21 or are a consequence of systemic responses to the DS pregnancy, logistic regression analysis was carried out. The results showed that the decreased TAS/TAC levels in maternal plasma (*P* < .05) and 8-OHdG, leptin, and adiponectin levels in amniotic fluid (*P* < .05) are directly related to the T21 occurrence (Tables 1 and 2).

**Table 1.** The trisomy 21 regression analysis in plasma

Parameter	B	SE	*P*	OR (95% CI)
Oxidative stress markers				
SOD	−35.254	17.569	**.0197**	<0.001 (0.001-0.007)
TAC	−0.029	0.001	**.0008**	0.97 (0.954-0.987)
TOC	−0.001	<0.001	.3975	1.0 (0.997-1.001)
8-OHdG	0.0003	0.001	.8116	0.85 (0.998-1.003)
NFκB	−0.069	2.214	.9753	0.94 (0.007-6.033)
FOXO	−2.142	1.869	.2518	0.12 (0.001-3.079)
SIRT 1	1.349	1.212	.2658	3.85 (0.455-66.041)
Metabolic-related markers				
Leptin	−0.070	0.037	.0572	0.93 (0.86-0.998)
Adiponectin	−0.002	0.031	.9523	1.0 (0.937-1.063)

Bold values indicate statistical significance at *P* < .05.

Abbreviations: CI, confidence interval; OR, odds ratio; PANEL, SOD + TAC + Leptin; SOD, superoxide dismutase; TAC, total antioxidant capacity; VIF, variance inflation factor.

**Table 2.** The trisomy 21 regression analysis in amniotic fluid

Parameter	B	SE	*P*	OR (95% CI)
Oxidative stress markers				
SOD	−5.231	3.736	**.0448**	0.23 (0.001-0.321)
TAC	0.009	0.009	.3846	1.01 (−0.008-0.027)
TOC	0.019	0.006	.0566	1.02 (1.006-1.049)
8-OHdG	0.038	0.001	**.001**	1.04 (1.01-1.09)
NFκB	−1.835	2.701	.497	0.16 (0.001-27.23)
FOXO	−3.271	2.037	.1084	0.04 (0.001-1.318)
SIRT 1	−0.476	0.484	.3253	0.62 (0.198-1.433)
Metabolic-related markers				
Leptin	−0.236	0.098	**.0163**	0.79 (0.630-0.930)
Adiponectin	0.634	0.180	**.0004**	1.89 (1.416-2.961)

Bold values indicate statistical significance at *P* < .05.

Abbreviations: 8-OHdG, 8-hydroxy-2′-deoxyguanosine; CI, confidence interval; FOXO, forkhead box protein O; NFκB, nuclear factor kappa-light-chain-enhancer of activated B cells; OR, odds ratio; SIRT1, sirtuin 1; SOD, superoxide dismutase; TAC, total antioxidant capacity; TOC, total oxidative capacity.

#### Regression panels

**Figure 7.**
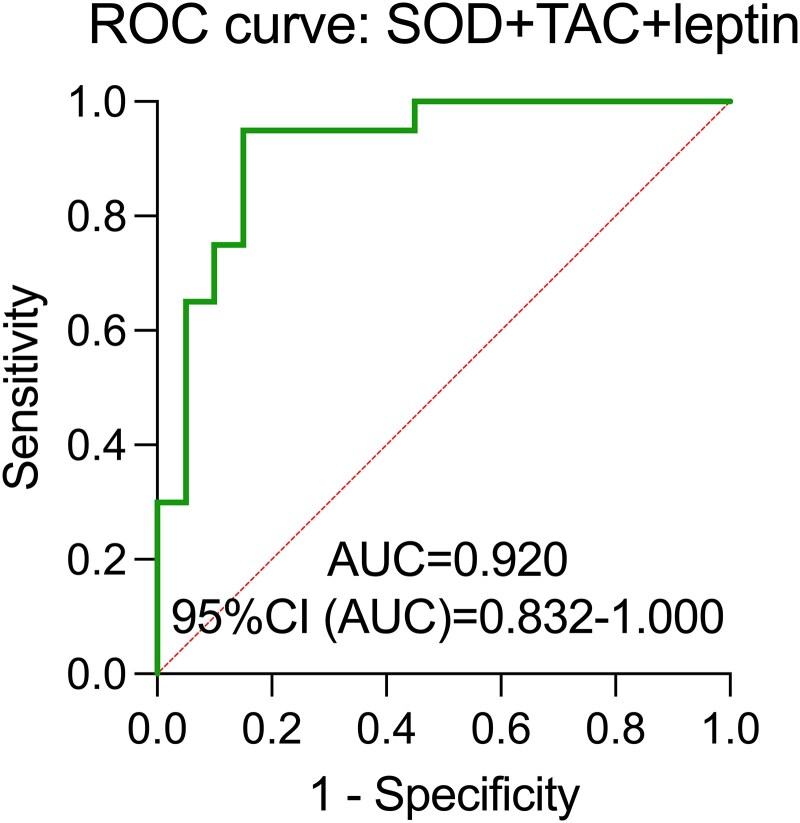
Regression plasma panels, with *P* < .05 considered significant.

Abbreviations: AUC, area under the ROC curve; ROC, receiver operating characteristic; SOD, superoxide dismutase; TAC, total antioxidant capacity.

The logistic regression model, a commonly used supervised learning technique for binary classification tasks, was utilized to increase the clinical utility of biomarkers and develop a noninvasive screening signature for T21 detection. Following, a logistic regression model using combination of SOD and TAC with leptin assessment yielded the highest clinical utility in T21 differentiation from healthy pregnancy (AUC = 0.920, *P* < .001). This predictive model was characterized by the lowest corrected Akaike information criterion (37.27) and variance inflation factor values (SOD = 1.037, TAC = 1.037, and leptin = 1.001, respectively) with predicted power of 85% (Fig. 7, Table 3). Moreover, the Hosmer-Lemeshow test proved the correction of created panel (*P* > .57).

**Table 3.** The trisomy 21 regression screening panel analysis

Parameter	B	SE	*P*	OR (95% CI)	VIF	R2 with other variables
PANEL	21.33	6.51	**.0010**	(10.56-36.90)	—	—
SOD	−31.45	17.95	.0798	2 185e-014 (0.001-0.321)	1.037	0.03601
TAC	−0.03781	0.01	**.0021**	0.9629 (−0.008-0.027)	1.037	0.03560
Leptin	−0.1253	0.05	**.0151**	0.8823 (1.006-1.049)	1.001	0.00057

Bold values indicate statistical significance at *P* < .05.

Abbreviations: CI, confidence interval; OR, odds ratio; SOD, superoxide dismutase; TAC, total antioxidant capacity; VIF, variance inflation factor, PANEL = SOD + TAC + Leptin.

### Genetic Pattern Related to T21

**Figure 8.**
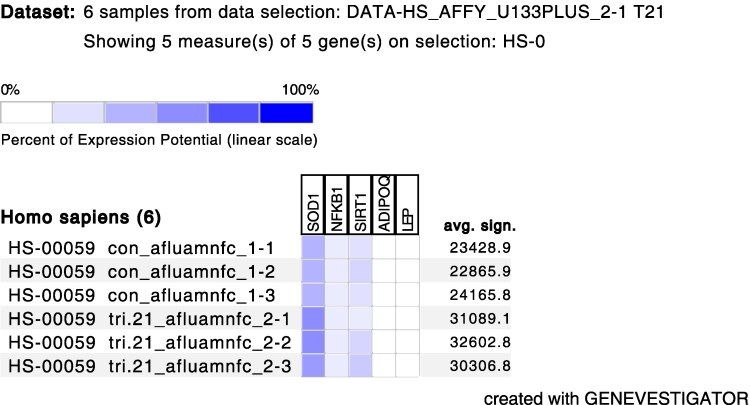
The gene expression profiling related to fetal trisomy 21 occurrence. The intensity of the color increases with the increase in expression.

To further elucidate the lack of association in the odds ratio analysis, we conducted an in-depth exploration of genetic databases. This analysis aimed to determine whether the identified proteins were linked to gene expression patterns, particularly focusing on next-generation sequencing data obtained from single-cell amniotic fluid samples. Exploring the cellular response to oxidative stress in T21 revealed that the condition is associated with the overexpression of superoxide dismutase 1 (*SOD1*) and sirtuin 1 (*SIRT1*) genes, while the expression of nuclear factor kappa B subunit 1 (*NFKB1*), adiponectin (*ADIPOQ*), and leptin *LEP* was found to be decreased, as detailed in Fig. 8.

## Discussion

Current literature consistently emphasizes the crucial role of oxidative stress in the pathogenesis of T21, primarily attributed to the dysregulation of gene expression induced by the presence of an additional copy of the chromosome 21 (33-35). Our previous studies identified increased levels of oxidative stress-related markers associated with fetal T21 pregnancy and demonstrated disruptions in oxidative stress and metabolic processes within the maternal compartment (6, 16, 17, 26). However, the precise biochemical pathways underlying these disruptions remain unclear, necessitating further investigation into specific markers. Thus, we analyzed key biochemical markers associated with oxidative stress and metabolic dysregulation in T21 pregnancies, focusing on TOC, TAC, 8-OHdG, SOD1, SIRT1, leptin, and adiponectin in maternal plasma and amniotic fluid, where a significant reduction in maternal plasma SOD and TAC levels in T21 pregnancies was observed. Furthermore, lower maternal plasma levels of FOXO and leptin indicate further impairment in the regulation of oxidative stress and metabolic processes (36-39). These results suggest a compromised antioxidant defense system within the maternal compartment, likely driven by the presence of the T21 fetus, which imposes greater oxidative stress on the maternal system (40, 41). This oxidative imbalance is further supported by elevated TOC and 8-OHdG levels in the amniotic fluid, confirming that T21 occurrence contributes to elevated oxidative stress and DNA damage, affecting both fetal and maternal compartments. Additionally, elevated SOD and TAC levels in the fetal compartment suggest an active response to oxidative stress, whereas this compensatory mechanism is not observed in the maternal compartment, where SOD and TAC are reduced. This discrepancy may indicate a maternal-to-fetal transfer of antioxidants, contributing to maternal antioxidant depletion while supporting fetal oxidative stress defence, ultimately highlighting a fetal-driven metabolic burden in T21 pregnancies. Given this imbalance, identifying specific maternal antioxidant deficiencies could enable the implementation of personalized medicine approaches, tailoring supplementation or treatment strategies to address individual metabolic needs (42). Moreover, the observed increase in adiponectin and leptin within amniotic fluid implies altered fetal metabolic regulation, with the disruption of leptin levels likely attributable to placental dysfunction or impaired fetal-maternal signaling rather than direct genetic regulation or increased T21 fetal production (43, 44). These deregulations may be addressed by an increase in insulin resistance within the fetal compartment, as indicated by Johnson et al, who found that neonates with T21 were 7 times more likely to develop permanent neonatal diabetes (45). Conversely, SIRT1 levels were significantly decreased in amniotic fluid. Literature data indicated that reduced SIRT1 levels were also observed in type 2 diabetes mellitus, which may confirm increasing insulin resistance in fetal T21 (46-48). Interestingly, in type 2 diabetes mellitus, decreased SIRT1 was correlated with increased DNA damage, as also observed in our study through the elevated 8-OhdG amniotic fluid (49), confirming ongoing oxidative DNA damage (50). Supporting this finding, the negative correlations between maternal plasma SOD activity, plasma TAC, and amniotic fluid adiponectin levels suggest that fetal insulin resistance depletes maternal antioxidants, increasing oxidative stress and reinforcing the systemic metabolic burden in T21 pregnancies (3, 7). Conversely, positive correlations between plasma TAC, plasma leptin, and amniotic fluid leptin levels, as well as between plasma leptin and FOXO, indicate significant interactions between maternal and fetal oxidative and metabolic dysregulations. Leptin plays a crucial role in fetal development by regulating energy metabolism, supporting neurological development, influencing growth processes, interacting with other hormones, and modulating the immune system. In T21, decreased leptin levels may impair neuronal activity, potentially leading to obesity and related health complications in individuals with DS. These effects likely result from maternal programming, disruptions in protein levels during fetal development, and the diffusion of these proteins into the amniotic fluid (38, 51, 52). These findings suggest that these factors may be correlated with T21 occurrence and could serve as potential risk indicators for the condition.

To further clarify the origins of these metabolic disturbances, we aimed to mathematically assess the transport of these biomarkers between maternal and fetal compartments. The AF/P ratio was significantly elevated for SOD, TAC, TOC, and adiponectin, indicating a predominantly fetal origin of these markers. If T21 fetuses predominantly produce SOD, TAC, TOC, and adiponectin, it suggests that the fetal compartment is actively counteracting oxidative stress (26). Previous studies have generally assumed that increased oxidative stress in T21 pregnancies arises equally in both the maternal and fetal compartments (53-55). However, the present findings challenge this assumption, indicating that fetal oxidative stress contributes to and exacerbates maternal redox imbalance, rather than originating from the maternal compartment. This depletion may be driven by placental transport mechanisms, which redirect key antioxidants such as SOD and TAC toward the fetus, prioritizing fetal protection at the cost of maternal redox homeostasis. Consequently, rather than reflecting a fetal adaptation intended to mitigate maternal oxidative stress, this response likely represents a consequence of the systemic oxidative burden associated with trisomy 21 pregnancies. These findings suggest that previous interpretations of oxidative stress in T21 pregnancies may have overlooked the bidirectional nature of fetal-maternal oxidative interactions. Furthermore, the reduced SIRT1 ratio supports these findings, reinforcing the concept of a fetal-driven metabolic imbalance affecting both compartments. The decreased SIRT1 ratio suggests compromised metabolic regulation, potentially contributing to increased insulin resistance and impaired oxidative stress control in the fetal compartment (53, 56). This imbalance may further exacerbate maternal oxidative stress, strengthening the notion that T21 pregnancies impose a bidirectional metabolic strain on both the mother and the fetus. Moreover, the negative correlation between plasma NFκB and SIRT1 levels underscores the complex interplay between inflammation and cellular stress responses in T21 pregnancies, further indicating that the reduced SIRT1 ratio reflects systemic metabolic dysfunction rather than an isolated fetal adaptation. This interplay may suggest that within the maternal compartment, high NFκB activity attempts to activate additional mechanisms to counteract stress; however, these efforts are ineffective due to the low activity of SIRT1, leading to insufficient stress resistance and impaired metabolic regulation (57, 58). This can impair growth and development, raising the risk of congenital anomalies and synaptic plasticity (59). As demonstrated by studies on fetal programming, which also encompass prenatal programming, environmental factors experienced during fetal development are crucial in determining health trajectories throughout an individual's lifespan (60, 61). This theory underscores the intricate interplay between maternal and fetal physiology, highlighting how the maternal environment significantly influences fetal development and the risk of metabolic-related diseases (62).

To gain deeper insight into the underlying causes of these metabolic disturbances, we performed logistic regression analysis to examine the relationship between these markers and T21. The logistic regression results indicated that decreased TAC levels in maternal plasma (*P* < .05) and elevated levels of 8-OHdG, leptin, and adiponectin in amniotic fluid (*P* < .05) are directly associated with the occurrence of T21. Furthermore, the transcriptomic validation revealed the expression of *SOD* and *SIRT* genes in amniotic cells based on single-cell RNA analysis data, suggesting that the increased ratio of these proteins indeed may result from production within the fetal compartment. The lack of expression of *NFKB1*, *ADIPOQ*, and *LEP* in amniotic fluid suggests that the observed biochemical alterations result from systemic physiological responses rather than being solely dictated by the extra chromosome 21. This highlights the role of fetal-maternal metabolic interactions, placental function, and oxidative stress adaptation in shaping the biochemical environment in T21 pregnancies. These factors may contribute to compensatory mechanisms or disruptions in protein synthesis, transport, and regulation, emphasizing the complexity of T21-related metabolic dysregulation beyond direct genetic determinants. Therefore, the functional analysis of *SOD1, SIRT1, NFKB1, ADIPOQ*, and *LEP* gene expression would provide critical insights into the T21 pathophysiology, as indicated by other authors (11, 15, 63-65). However, the most intriguing finding of this study was that elevated concentrations of the studied parameters in maternal plasma did not consistently result in higher levels in the amniotic fluid, indicating a complex relationship beyond simple diffusion and dysregulation of gene expression induced by the presence of an additional copy of the chromosome 21. Additionally, the concentrations of other markers varied between maternal and fetal compartments, suggesting that the T21 pattern may facilitate the transfer of proteins from maternal circulation to the placenta, compensating for impaired fetal synthesis. However, the gene expression levels did not always reflect the T21 origin and protein concentrations following potential diffusion to the amniotic fluid from the maternal compartment as indicated by AF/P ratio evaluations. To fully understand this association, further studies on the transfer mechanisms between maternal and fetal compartments are needed.

Nevertheless, a significant strength of this study is the simultaneous examination of both maternal and fetal components with the screening potential of oxidative stress markers in T21-affected pregnancies, allowing for a comprehensive assessment of their clinical significance (16, 26). The potential panel of maternal plasma SOD and TAC with leptin combined assessment in T21 prenatal screening was demonstrated, with a notable AUC of 0.920, comparable to the accuracy of free fetal DNA measurement with the confidence interval being assumed from 87.66% to 100.00% (66) and with the odds ratio for T21 in successful vs failed tests equal 98% (67). The widely used noninvasive prenatal test for T21 risk, integrating ultrasound markers with pregnancy-associated plasma protein A (PAPP-A) and serum-free human chorionic gonadotropin (B-HCG), demonstrates a commendable 93% accuracy. Additionally, individual diagnostic efficacy in maternal plasma has been established as follows: AUC for PAPP-A = 0.777, AUC for B-HCG = 0.668, and AUC for combined PAPP-A + B-HCG = 0.8533 (68).

Nevertheless, this study is subject to several limitations. First, the relatively small sample size (n = 40) may constrain the generalizability of the results and highlights the need for validation in larger, independent cohorts. Second, while transcriptomic validation was employed to investigate the potential origin of biochemical markers, it relied on publicly accessible datasets from the Genevestigator platform rather than on primary gene expression analyses conducted within the study population. These external datasets may differ in terms of population characteristics, data quality, and analytical methodology, potentially influencing the interpretation of results. Lastly, the cross-sectional and observational nature of the study precludes the establishment of causal relationships. Due to the limited size of the experimental group, it is crucial to conduct further assessments and validate the findings using a larger cohort to confirm the screening efficacy of the oxidative stress and energy metabolism parameters studied.

Despite these limitations, the results of this study align with existing research, demonstrating a consistent pattern of oxidative stress and related metabolic disturbances in T21 pregnancies (3, 9, 40, 69, 70). In the study performed by Perluigi et al, elevated levels of oxidative stress, evidenced by increased protein oxidation, lipid peroxidation, reduced glutathione, and thioredoxin levels, along with the induction of the heat shock protein response were observed among T21 pregnancy group (9). These findings suggest that oxidative damage is an early event in the pathogenesis of T21 and may contribute to the emergence of deleterious DS phenotypes, such as abnormal development and neuropathology resembling Alzheimer's disease (27). Similar studies conducted by Perone et al reported a 9-fold increase in isoprostane concentrations, a novel marker of free radical-catalyzed lipid peroxidation, in the amniotic fluid T21 (40). On the other hand, in a study conducted by Hedley et al, amniotic fluid leptin levels were examined in T21 pregnancies, showing no significant differences between studied groups (71). It is important to note that this analysis was conducted during early pregnancy, specifically between 8 and 14 weeks' gestation. During this period, variations in leptin concentrations may not be readily detectable, which could impact the misassessment of its role in fetal development. Consequently, Radunovic et al investigated leptin levels in plasma and fetal umbilical blood samples obtained during cordocentesis in both T21 and healthy pregnancies, after the 17th week of gestation. In the control group, fetal leptin levels were noticeably lower than maternal levels but displayed a consistent upward trajectory throughout gestation. In contrast, T21 pregnancies exhibited a significant reduction in fetal leptin levels, in both maternal and fetal compartments (72). This finding is consistent with the results of our study, which analyzed plasma and amniotic fluid samples obtained from T21 pregnancies. Nevertheless, our findings indicate that oxidative stress originates from T21 itself, with the fetus actively contributing to the imbalance by depleting maternal antioxidant resources, while also being a consequence of the condition, as suggested by differences in the analyzed metabolites (6). Therefore, a higher susceptibility to oxidative damage and metabolic imbalances in the developing fetus, highlighting the importance of monitoring and increasing antioxidant defense in T21 pregnancies to improve outcomes and support fetal development (14, 35). Apigenin exhibits multiple biological effects, including reducing oxidative stress, inhibiting NFκB–mediated inflammation, and promoting neurogenesis by upregulating the expression of genes associated with neural proliferation and differentiation, such as marker of proliferation ki-67 (*MKI67*), nestin (*NES*), SRY-BOX transcription factor 2 (*SOX2*), and paired box 6 (*PAX6*). These effects may counteract some of the key neurodevelopmental impairments observed in DS fetuses, suggesting a prenatal window for therapeutic intervention (73). In addition to apigenin, metformin emerges as another promising candidate for investigation in T21 pregnancies. Metformin is known to improve insulin sensitivity, reduce oxidative stress, and modulate mitochondrial function, mechanisms that align with the metabolic disturbances observed in T21 fetuses. Furthermore, metformin has been shown to activate AMPK and SIRT1, which could mitigate fetal-driven maternal antioxidant depletion and enhance cellular stress resistance. Given the established safety profile of metformin during pregnancy, its potential role in improving metabolic homeostasis in T21 fetuses and reducing maternal oxidative burden warrants further investigation (6, 17, 74-76).

## Conclusions

This study demonstrates significant oxidative stress and metabolic disturbances in T21 pregnancies, with distinct differences observed between maternal plasma and amniotic fluid. Maternal plasma showed decreased TAC, SOD, FOXO, and leptin levels, while amniotic fluid exhibited lower FOXO and leptin but higher SOD, TAC, TOC, adiponectin, and 8-OHdG levels. The AF/P ratio was significantly increased for SOD, TAC, TOC, and adiponectin, indicating a fetal origin of these markers, whereas the reduction in SIRT1 suggests impaired fetal oxidative regulation. However, the altered leptin levels in both compartments suggest placental dysfunction or disrupted fetal-maternal signaling, rather than direct fetal synthesis, as no significant changes were observed in the AF/P ratio. Nevertheless, logistic regression analysis confirmed that decreased TAC in maternal plasma and elevated 8-OHdG, leptin, and adiponectin in amniotic fluid are directly associated with T21 pregnancies. Additionally, genetic validation did not support a direct link between leptin and adiponectin with T21 gene expression, suggesting that metabolic alterations arise from systemic physiological responses, more likely driven by increased insulin resistance in the fetal compartment rather than a direct genetic influence. Thus, the plasma combination of SOD, TAC, and leptin, analyzed using regression with panel modeling, demonstrated the highest clinical utility for distinguishing T21 pregnancies (AUC = 0.92, *P* < .001). These findings support the potential of multimarker profiling for noninvasive prenatal screening and highlight the need for further investigation into the metabolic interplay between the fetus, placenta, and maternal system in T21 pregnancies.

## Data Availability

The datasets used and/or analysed during the current study available from the corresponding author on reasonable request.
